# Effects of secukinumab on bone mineral density and bone turnover biomarkers in patients with ankylosing spondylitis: 2-year data from a phase 3 study, MEASURE 1

**DOI:** 10.1186/s12891-021-04930-1

**Published:** 2021-12-13

**Authors:** Jürgen Braun, Bjoern Buehring, Xenofon Baraliakos, Lianne S. Gensler, Brian Porter, Erhard Quebe-Fehling, Sibylle Haemmerle

**Affiliations:** 1 Rheumazentrum Ruhrgebiet Herne, Ruhr-University, Bochum, Germany; 2 University of California, San Francisco, USA; 3 Novartis Pharmaceuticals Corporation, East Hanover, USA; 4 Novartis Pharma AG, Basel, Switzerland

**Keywords:** Ankylosing spondylitis, Secukinumab, Bone mineral density, Bone turnover biomarkers, Osteoporosis, Osteopenia, Vertebral fractures

## Abstract

**Background:**

Axial spondyloarthritis including ankylosing spondylitis (AS) is characterized by chronic inflammation and new bone formation in the axial skeleton. On the other hand, bone loss, osteoporosis and an increased risk of vertebral fractures is known to frequently occur in AS. In the MEASURE 1 study, the clinically efficacious interleukin-17A inhibitor secukinumab was shown to have limited radiographic progression through 4 years in patients with active AS. Here we present a post hoc analysis to evaluate the effect of secukinumab on bone mineral density (BMD) and bone turnover biomarkers over 2 years in this study.

**Methods:**

BMD was measured by dual-energy X-ray absorptiometry at the lumbar spine, total hip, and femoral neck. Spinal radiographs performed at baseline and Week 104 were assessed by modified Stoke Ankylosing Spondylitis Spinal Score (mSASSS) and analyzed in relation to BMD change, considering baseline syndesmophytes. Bone turnover biomarkers were assessed at baseline and at Weeks 52 or 104.

**Results:**

Among 104 patients included in this analysis, 66% were male, with a mean (SD) age of 40.4 (12.3) years. In postmenopausal women and men ≥50 years of age (T-score), the proportion of patients having normal BMD at baseline and Week 104 were 54.5%/54.5% (lumbar spine), 31.6%/55.6% (total hip), and 42.1%/44.4% (femoral neck). Similarly, at baseline, the proportion of patients with osteopenia/osteoporosis was 31.8%/13.6% (lumbar spine), 57.9%/10.5% (total hip), 42.1%/15.8% (femoral neck), and 36.4%/9.1% (lumbar spine), 44.4%/0% (total hip) and 55.6%/0% (femoral neck) at Week 104, respectively. In premenopausal women and men < 50 years of age (Z-score), the proportion of patients having BMD below the expected range for age at baseline and Week 104 were 25.0%/21.2% (lumbar spine), 11.3%/17.8% (total hip), and 9.9%/8.9% (femoral neck). In relation to mSASSS change scores ≥2 over 2 years, the increase in lumbar spine BMD was not related to radiographic progression and syndesmophyte formation. No significant changes were observed in the bone turnover markers over time.

**Conclusion:**

The high proportion of AS patients with diminished BMD was confirmed in this study. An increase of BMD in the lumbar spine after 2 years of secukinumab treatment in patients with AS was found that was probably unrelated to radiographic progression. No relevant effects of secukinumab on bone turnover biomarkers were documented.

**Trial registration:**

MEASURE 1 (post hoc analysis) Clinicaltrials.gov, NCT01358175; Registered, 23 May 2011.

**Supplementary Information:**

The online version contains supplementary material available at 10.1186/s12891-021-04930-1.

## Background

Ankylosing spondylitis (AS) or radiographic axial spondyloarthritis (axSpA), is a chronic inflammatory disease that primarily affects the axial skeleton, including the sacroiliac joints and the vertebral column [1]. It is often characterized by inflammatory back pain and spinal stiffness leading to impaired physical function [2]. Extra-musculoskeletal manifestations such as psoriasis [3] and comorbidities such as osteoporosis can have substantial impact on quality of life in patients with AS [4, 5]. Structural bone damage and spinal radiographic progression in AS typically manifests by syndesmophyte formation and ankylosis [6, 7].

As a consequence of ongoing systemic inflammation, osteopenia or decreased bone mineral density (BMD) are common findings in patients with AS [8, 9]. Low BMD at the spine and hip is associated with an increased risk of fracture in patients with AS [10, 11]. The prevalence of osteoporosis in patients with AS is usually > 10% but varies according to age, disease duration, gender, the burden of inflammation and new bone formation, diet, vitamin D levels and immobility [12, 13]. Bone formation and loss of bone mineral content may occur in parallel in AS. The exact pathomechanisms leading to bone growth and loss, and the association between them is still unclear [5, 14, 15]. Due to significantly increased fracture risk an early diagnosis and treatment of osteoporosis is very important in patients with AS [12, 13, 16]. In addition to BMD assessment using dual-energy X-ray absorptiometry (DXA), biochemical markers of bone metabolism, known as bone turnover biomarkers, including markers of bone formation (growth), bone resorption (loss) and bone turnover regulators, can be useful diagnostic tools for the assessment of fracture risk in patients with AS [16, 17].

Biologics, such as interleukin-17 (IL-17) inhibitors and tumor necrosis factor inhibitors (TNFi), are recommended for treatment in patients with AS [18, 19]. In recently published studies, limited radiographic progression has been reported in patients with AS over long-term treatment with TNFi [20, 21]. However, majority of data on the effect of TNFi on BMD is inconclusive [21–24].

Secukinumab, a human monoclonal antibody that directly inhibits IL-17A, provided sustained improvement in patients with active AS in the pivotal Phase III MEASURE 1 study through 5 years [25, 26]. Data from this study reported a low mean progression of spinal radiographic change in secukinumab-treated patients through 4 years. However, the effect of secukinumab on BMD has not been explored yet [27–29]**.** Here, we present a post hoc analysis to evaluate the effect of secukinumab 150 mg (approved dose for AS) treatment over 2 years on BMD and bone turnover biomarkers in patients with AS from the MEASURE 1 study. A further analysis of BMD categories was performed on the following subgroups: postmenopausal women and men ≥50 years of age, and premenopausal women and men < 50 years of age.

## Methods

### Study design and patients

MEASURE 1 was a double-blind, randomized, placebo-controlled Phase III 2-year core study, with a 3-year extension study. The detailed study design, patients, methodology and statistical analyses have been described previously [25, 26]. Patients were initially randomized to receive intravenous (i.v.) secukinumab 10 mg/kg at baseline and at Weeks 2 and 4, followed by subcutaneous (s.c.) secukinumab 150 (IV → 150 mg) or 75 mg (IV → 75 mg) every 4 weeks thereafter. Placebo was given on the same i.v. to s.c. dosing schedule. Placebo patients were re-randomized to s.c. secukinumab 150 mg or 75 mg by Week 16 (Assessment of SpondyloArthritis International Society criteria 20 [ASAS 20] non-responders at Week 16) or Week 24 (ASAS 20 responders at Week 16). In the current post hoc analysis, data from patients originally randomized to secukinumab 150 mg (IV → 150 mg) were analyzed.

In brief, the key inclusion criteria included patients aged ≥18 years with active AS fulfilling the modified New York Criteria [30], with a Bath Ankylosing Spondylitis Disease Activity Index (BASDAI) score of ≥4 (on a 0–10 scale) [31] and a spinal pain score of ≥4 cm on a 10 cm visual analogue scale, despite treatment with nonsteroidal anti-inflammatory drugs. Patients who had taken not more than one previous TNFi with an inadequate response or intolerance were also allowed to enter the study. Key exclusion criteria were total spinal ankylosis, evidence of infection or cancer on chest radiography, active systemic infection within 2 weeks before baseline, and previous treatment with cell-depleting therapies or biologic agents other than TNFi agents.

This multicenter randomized clinical trial (RCT) was well designed and fulfilled the CONSORT statement checklist, which comprises a minimum standard of recommendations for reporting RCTs [32].

### Outcome measures

#### BMD assessment

BMD (g/cm^2^) was measured by DXA using Hologic or GE-Lunar scanners at the lumbar spine, total hip, and femoral neck. Sites were instructed to use the same DXA scanner throughout the trial. The BMD values were standardised using a conversion formula by the central imaging laboratory by doing cross validation. Quality assurance, cross-calibration adjustment, and data processing were done centrally by a certified imaging specialist.

#### T-score

The T-score is the standard deviation of BMD of an individual compared to the mean BMD of young (20–29 year-old) healthy adults. According to the World Health Organization, the T-score categorizes postmenopausal women and men aged ≥50 years as follows: normal BMD: T-score ≥ − 1.0; osteopenic: − 2.5 < T-score < − 1.0; and osteoporotic: T-score ≤ − 2.5 [33, 34].

The change from baseline in T-score was calculated at Weeks 52 and 104.

#### Z-score

The Z-score is the BMD standard deviation of the individual compared to the mean BMD of individuals of the same gender, age, and ethnicity. Z-score values, as far as available (not available for example, Asians), were added to the study database in the preparation of this post-hoc analysis. According to the International Society for Clinical Densitometry, the Z-score categorizes premenopausal women and men aged < 50 years as below the expected range for age with BMD ≤ − 2.0 and within the expected range for age with BMD > − 2.0 [35].

The change from baseline in Z-score was calculated at Weeks 52 and 104. The age-matched Z-scores for 3 skeletal sites (lumbar spine, total hip, and femoral neck) at Weeks 52 and 104 were also calculated.

#### Bone turnover biomarkers

Bone turnover biomarkers such as the anabolic or bone formation biomarkers [osteocalcin (μg/L), procollagen type 1 N-terminal propeptide (μg/L), procollagen type 1 carboxy-terminal peptide (μg/L), bone specific alkaline phosphatase (U/L)], the bone resorption biomarker type I collagen C-telopeptides (μg/L), and other biomarkers (bone turnover regulators) such as osteoprotegerin (pmol/L) and sclerostin (pmol/L) were assessed by a validated enzyme-linked immunosorbent assay (ELISA) method with a lower limit of quantification of 80 ng/mL. These bone turnover biomarkers reflect the rate of bone formation and bone resorption over the treatment phase [14, 16].

Serum samples for biomarker analysis were collected before 11 am after an overnight fast of at least 8 h at selected visits. Changes from baseline in bone turnover biomarkers were summarized at Weeks 52 and 104.

#### Radiographic assessment

The cervical and the lumbar spine radiographs (lateral view) were obtained at baseline and Week 104. Images were digitized centrally and to ensure blinding, patient identifiers and temporal indicators were removed [28]. Structural damage and radiographic progression was assessed by central readers using modified Stoke Ankylosing Spondylitis Spine Score (mSASSS; score range 0–72, higher score indicates greater structural damage) [36]. In radiographs scored using mSASSS, a lateral view of the anterior parts of lumbar spine is evaluated for the presence of squaring, erosions and/or sclerosis (1 point), syndesmophytes (2 points) and bridging syndesmophytes (3 points) [37]. mSASSS score was assessed in relation to BMD change, taking baseline syndesmophytes into consideration, with baseline syndesmophytes being robust predictors of radiographic progression [38]. Presence of baseline syndesmophytes was derived from mSASSS: according to the definition of levels of mSASSS, baseline syndesmophytes were considered present if a patient had at least one vertebra with a score of 2 or 3 [36, 39]. For the present analysis, ‘notable radiographic progression’ was set as an increase in mSASSS ≥2 units over 2 years of treatment.

### Statistical analysis

Analyses are post hoc and descriptive (data presented as observed). All analyses are presented for patients originally randomized to the secukinumab 150 mg group (IV → 150 mg). Analyses included patients with non-missing baseline BMD value and at least one post-baseline lumbar spine BMD value at Weeks 52 or 104 from the full analysis set. The baseline value is defined as the last observation on the day of or before the first dose of study drug.

To assess normalization of bone turnover markers, the course over time in the high and low quartile subgroups (at baseline) was explored by boxplots. A scatter plot was generated to evaluate the changes in lumbar spine BMD from baseline to Week 104 in relation to change in mSASSS from baseline to Week 104; patients with and without baseline syndesmophytes were represented by different symbols, and horizontal and vertical lines indicated the change in lumbar spine BMD of zero and change in mSASSS of 2, respectively, as depicted in Fig. 4.

## Results

### Patients

Baseline demographic and clinical characteristics in patients with AS from MEASURE 1 have been reported previously [25]. A total of 104 patients (originally randomized to secukinumab 150 mg) were included in this post hoc analysis; postmenopausal women and men ≥50 years of age, 21.1% (*n* = 22) and premenopausal women and men < 50 years of age, 78.8% (*n* = 82). Patients (*n* = 78) with a non-missing baseline BMD value and at least one post-baseline lumbar spine BMD value at Weeks 52 or 104 from the full analysis set were considered for further analysis of normal BMD, osteopenia, and osteoporosis. Among the 78 patients, 22 patients were postmenopausal women and men, and 56 patients were premenopausal women and men < 50 years of age.

Demographics and baseline BMD values for patients originally randomized to secukinumab 150 mg (IV → 150 mg) group (*N* = 104) are presented in Table 1. Within this population, 66.3% of patients were male, with a mean (SD) age at baseline of 40.4 (12.3) years. Overall, the mean time since diagnosis of AS was 6.4 years **(**Table 1). The mean (SD) baseline BMD (g/cm^2^) was 1.026 (0.2) at the lumbar spine, 0.902 (0.2) for total hip, and 0.819 (0.2) at the femoral neck at baseline. The mean baseline BMD T-scores in this population for lumbar spine (*N* = 104), total hip (*N* = 96) and femoral neck (*N* = 96) were − 0.6 (1.6), − 0.5 (1.2) and − 0.8 (1.1), respectively. The mean baseline BMD Z-scores for lumbar spine (*N* = 66), total hip (*N* = 84) and femoral neck (*N* = 84) were − 0.8 (1.6), − 0.6 (1.1) and − 0.6 (1.0), respectively (Table 1). In this analysis, taken together there were four patients with concomitant osteoporosis treatment including zoledronic acid and risedronate. However, due to small n numbers no further sub-group analysis was carried out in these patients.

**Table 1:** Demographics and Baseline BMD Values

Characteristic, mean (SD) unless otherwise specified	Secukinumab (***N*** = 104)
Age (years)	40.4 (12.3)
Male, n (%)	69 (66.3)
Caucasian, n (%)	61 (58.7)
BMI (kg/m^2^)	26.1 (4.8)
Time since AS diagnosis (years)	6.4 (7.1)
Lumbar spine BMD (g/cm^2^)	1.026 (0.2)
Hip BMD (g/cm^2^)	0.902 (0.2)
Femoral neck BMD (g/cm^2^)	0.819 (0.2)
Lumbar spine BMD T-Score	−0.6 (1.6)
Hip BMD T-Score	−0.5 (1.2)
Femoral neck BMD T-Score	−0.8 (1.1)
Lumbar spine BMD Z-Score	−0.8 (1.6)
Hip BMD Z-Score	−0.6 (1.1)
Femoral neck BMD Z-Score	−0.6 (1.0)

*BMI* body mass index, *BMD* bone mineral density, *N* number of patients with non-missing baseline BMD value and non-missing post-baseline lumbar spine BMD value at Week 52, *SD* standard deviation

### Efficacy

Efficacy data is reported only for patients originally randomized to secukinumab 150 mg (IV → 150 mg) group. The mean of individual BMD percent changes at Week 52 were 2.6% for lumbar spine, 0.9% for total hip, and 0.8% for femoral neck. Corresponding percent changes at Week 104 were 4.7% for lumbar spine, 0.5% for total hip, and 0.2% for femoral neck.

**Fig. 1:**
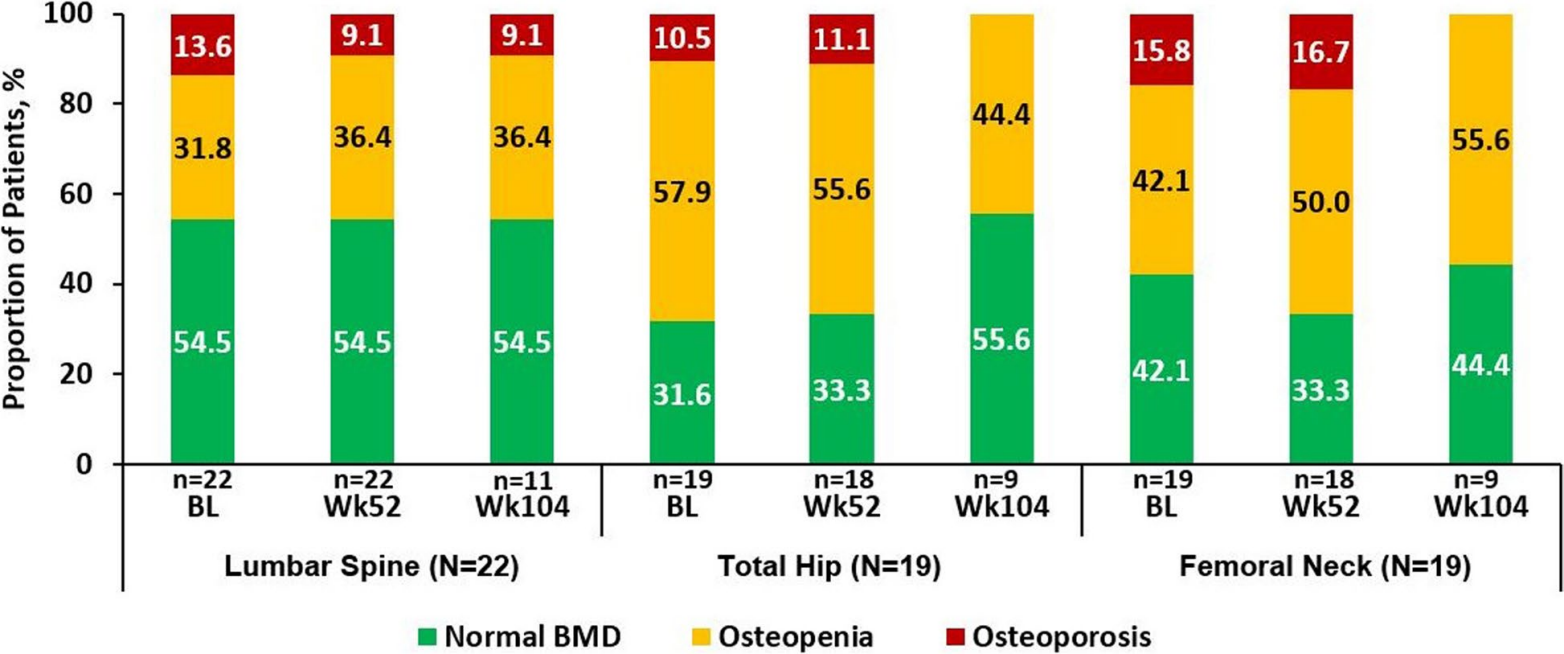
Normal BMD, Osteopenia, and Osteoporosis Rates in Postmenopausal Women and Men ≥50 years of age through Week 104. Data presented as observed. Normal BMD, T-score ≥ − 1.0; Osteopenia, − 1.0 > T-score > − 2.5; and Osteoporosis, T-score ≤ − 2.5. N, total number of patients; n, number of evaluable patients

**Fig. 2:**
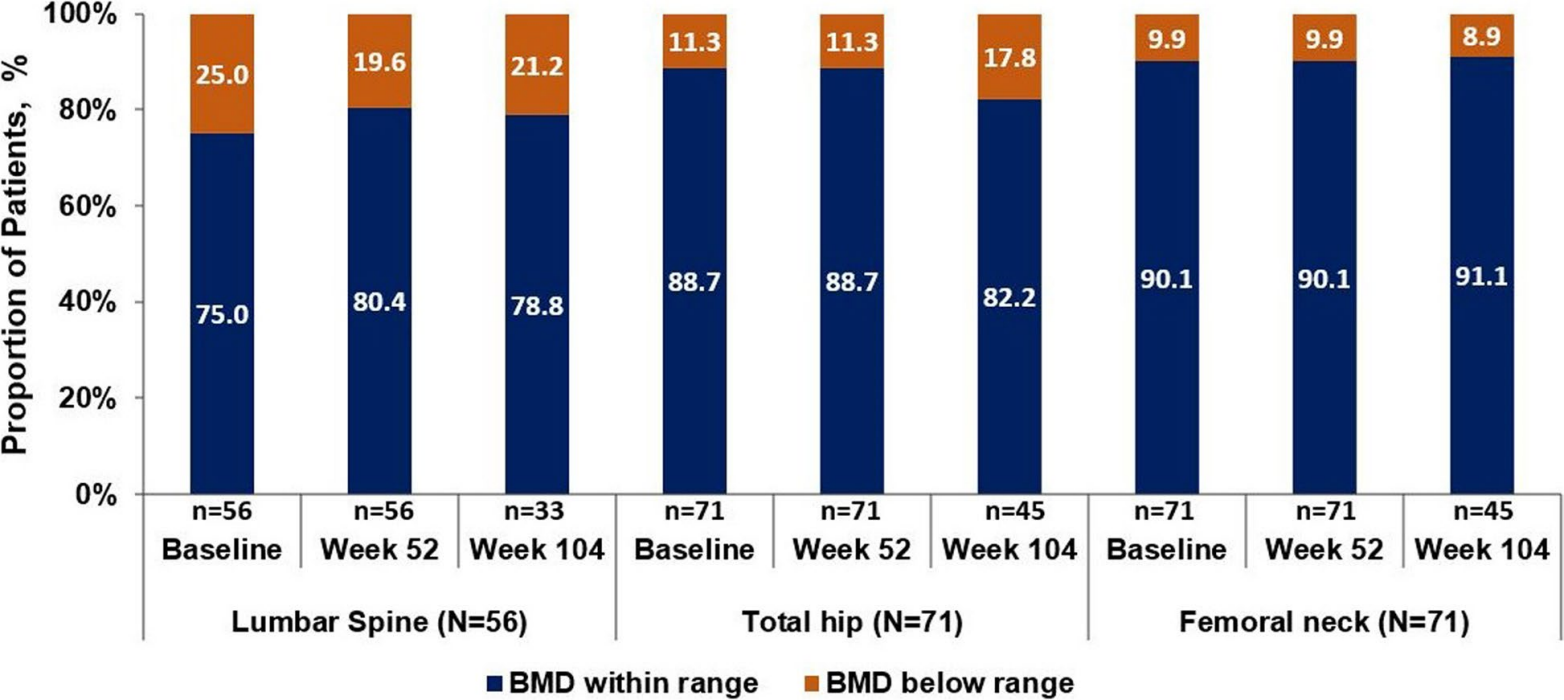
BMD Below and Within Expected Range in Premenopausal Women and Men < 50 Years of age through Week 104. Data presented as observed. BMD within the expected range for age (‘Within Range’) = Z score > − 2.0, and below the expected range for age (‘Below Range’) = Z score ≤ − 2.0. BMD, bone mineral density; N, total number of patients; n, number of evaluable patients

For postmenopausal women and men ≥50 years, the proportion of patients with normal BMD, osteopenia and osteoporosis was 54.5, 31.8 and 13.6%, respectively at baseline. The proportion of patients with normal BMD, osteopenia and osteoporosis remained consistent from baseline to Week 52 (54.5%/36.4%/9.1%) and Week 104 (54.5%/36.4%/9.1%) for the lumbar spine. A similar trend was observed for total hip and femoral neck where no considerable change in the number of patients in each group was observed over time (Fig. 1). In premenopausal women and men < 50 years of age, the proportion of patients having BMD below the expected range for age at baseline was 25.0, 11.3, and 9.9% for the lumbar spine, total hip, and femoral neck, respectively. Consistent with the results, the proportion of patients having BMD below the expected range for age was 21.2, 17.8, and 8.9% for the lumbar spine, total hip, and femoral neck, respectively at Week 104 (Fig. 2), indicating no substantial shift in the distribution of patients towards abnormal BMD through 2 years of treatment.

**Fig. 3:**
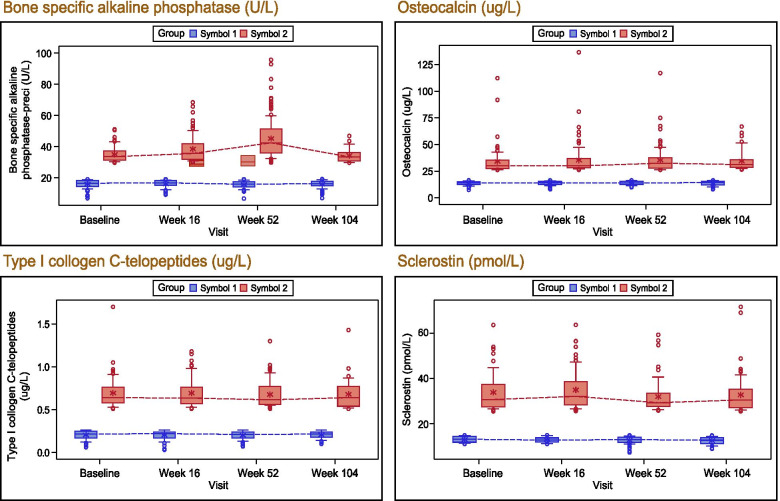
Bone turnover biomarkers course over time for highest and lowest quartile subgroups (at baseline). Symbol 1 – lowest quartile group of parameter at baseline. Symbol 2 – highest quartile group of parameter at baseline. For each subgroup: The box represents the upper and lower quartiles and the whiskers (10th and 90th percentiles) represents the minimum and maximum values. The center horizontal line represents the median and * represents the mean. Circles outside the boxes represent outliers

The time course in the high and low quartile subgroups (at baseline) for various bone turnover biomarkers are presented as boxplots in Fig. 3. Independent of high or low baseline values, bone turnover biomarkers remained in the same range overall, following a consistent pattern through 2 years of treatment. The mean changes from baseline in other biomarkers through Week 104 are listed in Table 2**.** Amongst the bone turnover (formation and resorption) markers, in active AS patients after 2 years of secukinumab therapy, no significant changes at Weeks 52 and 104 were observed.

**Table 2:** Mean Change from Baseline in Bone Turnover Biomarkers at Week 52 and 104

	Baseline, Mean (SD)	Week 52, Mean change (SD)	Week 104, Mean change (SD)
**Anabolic biomarkers**			
Osteocalcin (ug/L)	*n* = 99	*n* = 95	*n* = 71
	21.84 (7.56)	1.53 (6.58)	−0.39 (7.13)
Bone specific alkaline phosphatase (U/L)	*n* = 100	*n* = 96	*n* = 73
	25.01 (8.24)	9.07 (12.89)	−2.66 (6.54)
Procollagen type 1 N-terminal propeptide (ug/L)	n = 99	*n* = 95	*n* = 71
	50.76 (20.80)	−1.81 (15.98)	1.79 (21.97)
Procollagen-1 carboxy-terminal peptide (ug/L)	*n* = 100	*n* = 96	*n* = 73
	107.25 (46.83)	−5.73 (47.13)	10.04 (51.53)
**Bone resorption biomarkers**			
Type I collagen C-telopeptides (ug/L)	*n* = 99	*n* = 94	*n* = 71
	0.42 (0.20)	−0.01 (0.15)	−0.03 (0.14)
**Other biomarkers**			
Sclerostin (pmol/L)	*n* = 73	*n* = 63	*n* = 52
	21.66 (8.17)	0.05 (6.90)	2.38 (6.52)
Osteoprotegerin (pmol/L)	*n* = 98	*n* = 93	*n* = 71
	4.13 (1.50)	−0.91 (1.20)	0.49 (1.53)

Data presented as observed

Analysis included total number of patients who had baseline BMD plus at least one post-baseline lumbar spine BMD value (at Week 52 or 104)

*n* number of evaluable patients for each parameter at a particular visit, *SD* standard deviation

**Fig. 4:**
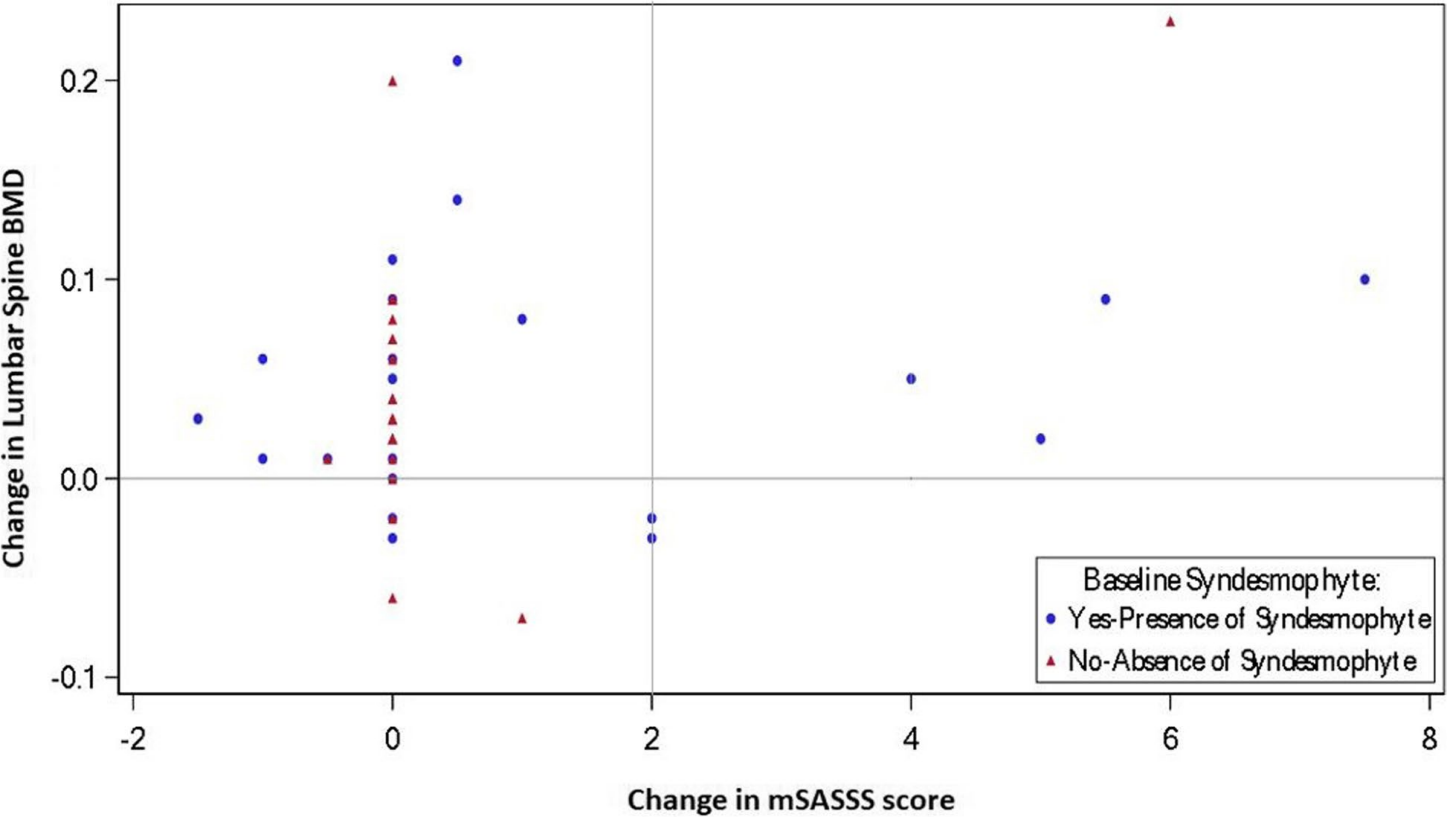
Scatter plot of change in lumbar spine BMD and mSASSS in patients with/without baseline syndesmophytes**.** BMD, bone mineral density; mSASSS, modified Stoke Ankylosing Spondylitis Spine Score. Horizontal line indicates change in the lumbar spine BMD of zero, and vertical line is an indicator of change in mSASSS of 2

The changes in the lumbar spine BMD from baseline to Week 104 in relation to change in mSASSS from baseline to Week 104 for patients with or without baseline syndesmophytes are presented in the form of a scatter plot to assess an increase in BMD in relation to radiographic progression (Fig. 4). A higher change in mSASSS score (≥ 2) and a higher probability for disease progression was observed in patients with baseline syndesmophytes compared to patients without baseline syndesmophytes.

Further, there was no notable change from baseline in mSASSS score (≥2) and no radiographic progression over time was observed in secukinumab-treated patients. As such, no correlation was seen between the increase in lumbar spine BMD and radiographic progression (syndesmophyte formation).

## Discussion

The effect of secukinumab treatment in patients with AS from the MEASURE 1 study through 5 years have been reported previously [25, 26]. Almost 45% of patients included in the MEASURE 1 study originally randomized to secukinumab 150 mg had a low BMD at the anteroposterior lumbar spine L1–L4 and almost 58% at the femoral neck, with osteoporotic values in more than 10% of patients (postmenopausal women and men ≥50 years), consistent with previous studies [5, 40, 41]. The proportion of patients with low BMD in premenopausal women and men < 50 years of age was 25% at the lumbar spine.

To the best of our knowledge, this post hoc analysis in secukinumab 150 mg (IV → 150 mg) group is the first study assessing the effects of an IL-17 inhibitor on BMD at different sites and in relation to radiographic progression, in combination with the evaluation of bone turnover markers. According to the demographic and baseline clinical characteristics, the assessment of BMD was analyzed based on T-score and Z-score due to the heterogeneity of the study population.

Increase in BMD from baseline in the spine (*P* < 0.001) and hip (*P* = 0.040) at Week 102 following treatment with the TNFi infliximab (ASSERT trial) have been previously reported in patients with ankylosing spondylitis [42]. In this post hoc analysis, BMD was maintained or increased over 2 years in both men and women treated with secukinumab (an IL-17A inhibitor), with a pronounced increase observed in the lumbar spine BMD, based on the mean of individual BMD percent changes. Thus, as seen with TNFi, secukinumab preserves BMD in patients with AS [24, 43]. However, the measurement of lumbar spine BMD with DXA in patients with AS is often problematic because BMD is falsely elevated due to bone proliferation or new bone formation, which is a characteristic feature of AS [44].

In MEASURE 1, low mean progression of spinal radiographic change at Week 104 was demonstrated with secukinumab [28, 29]. In the current post hoc analysis, the scatter plot showed no notable change or comparatively low change in mSASSS score through 2 years, despite the increase in BMD at the lumbar spine. Most of the patients who had a change in lumber spine BMD did not have a change in mSASSS. Hence, the increase in bone density/BMD was not due to increase in mSASSS score which is considered as a new bone formation due to disease worsening. This observation suggests that there is no association between lumbar spine BMD increase and syndesmophytes formation, as measured by mSASSS score and further substantiating the radiographic data reported over 2 years in the MEASURE 1 core study. Hence, a false elevation of BMD values based on syndesmophyte formation is not suggested from this analysis.

The prevalence of syndesmophytes is related to new bone formation, as is the case for BMD increase. However, syndesmophytes are the result of vertebra fusion (bridging of intervertebral spaces) with reduced bone quality than normal. The altered biomechanics and bone quality are thought to be responsible for the increased risk of vertebral fractures in patients with AS. Any increase in DXA measured BMD, which is not accompanied by an increase in radiographic progression, could be interpreted as evidence for improved bone quality and reduced fracture risk. However, other factors such as aortic calcifications, progressive degenerative changes in the lumbar spine not assessed in the mSASSS, osteoblastic lesions, or new vertebral fractures can also lead to increased BMD but would not improve bone quality or fracture risk. Only more advanced imaging analyses including quantitative computed tomography (QCT) or finite element analysis and/or prospective trials powered for vertebral fracture reduction could examine the hypothesis that the observed improvement in DXA measured BMD results in better bone strength and reduced fracture risk.

Bone turnover biomarkers, released during bone remodeling process, including bone formation and resorption, could have the potential to provide prognostic information on fracture risk to supplement radiographic measures of bone mass [45]. However, measurement of bone turnover biomarkers is often challenging as these markers are influenced by several pre-analytical and disease-related confounding factors (e.g., time of day when sampled, underlying renal function) [17]. As the MEASURE 1 study was not powered to detect differences in these often highly variable biomarkers, these results need to be interpreted with caution. It was observed that independent of higher and lower baseline values, bone turnover biomarkers (anabolic, bone resorption and other biomarkers) remained in the same range overall, following a consistent pattern through 2 years of secukinumab treatment. These results indicated no detrimental effect of secukinumab treatment on the bone metabolism.

Further limitations of this analysis include the unavailability of individual mSASSS data for cervical and lumbar spine, lack of a control group and a limited sample size. Any differences in BMD, T-score, or Z-score at the lumbar spine in patients with and without radiographic spinal progression was not analysed as a part of this analysis due to small patient numbers. The lack of vertebral fracture assessment and correlation analysis for bone turnover markers with BMD further limits the evaluation of BMD and bone turnover biomarker changes in relation to bone quality, as well as the overall interpretation of the findings.

## Conclusions

The results of this post hoc analysis from the MEASURE 1 study showed that after 2 years of treatment with secukinumab in patients with AS, BMD of the lumbar spine, femoral neck and total hip was stable or increased, with the most striking findings at the lumbar spine. Importantly, these increases in BMD were not correlated with radiographic mSASSS progression or biomarker changes.

## Supplementary Information

**Additional file 1 **: **Table S1.** List of ethical approval reference numbers for each participating center (MEASURE 1 study).

## Data Availability

The datasets generated and/or analysed during the current study are not publicly available. Novartis is committed to sharing with qualified external researchers’ access to patient-level data and supporting clinical documents from eligible studies. These requests are reviewed and approved based on scientific merit. All data provided are anonymized to respect the privacy of patients who have participated in the study in line with applicable laws and regulations. The data may be requested from the corresponding author of the manuscript.

## Abbreviations

AS: Ankylosing Spondylitis
axSpA: Axial Spondyloarthritis
BASDAI: Bath Ankylosing Spondylitis Disease Activity Index
BMD: Bone Mineral Density
DXA: Dual-energy X-ray Absorptiometry
IL: Interleukin
i.v.: Intravenous
mSASSS: Modified Stoke Ankylosing Spondylitis Spine Score
QCT: Quantitative computed tomography
s.c.: Subcutaneous
SD: Standard Deviation
TNFi: Tumor necrosis factor inhibitors
